# Transorbital endoscopic approach for repair of a lateral sphenoid sinus cerebrospinal fluid leak

**DOI:** 10.3171/2025.1.FOCVID24207

**Published:** 2025-04-01

**Authors:** Nader Delavari, Jesse D. Lawrence, Jonathan Tangsrivimol, Mattia Testa, Kyle J. Godfrey, Theodore H. Schwartz

**Affiliations:** 1 Department of Neurosurgery, NewYork-Presbyterian Weill Cornell, New York, New York;; 2 Department of Neurosurgery, Chulabhorn Hospital, Chulabhorn Royal Academy, Bangkok, Thailand;; 3 Department of Neurosurgery, University of Verona, Italy;; 4 Department of Ophthalmology, NewYork-Presbyterian Weill Cornell, New York, New York; and; 5 Scarsdale, New York

**Keywords:** transorbital approach, transpalpebral approach, sphenoid sinus, encephalocele, middle fossa, rhinorrhea

## Abstract

The transorbital craniotomy represents a minimally invasive approach to the mesial structures of the middle fossa. This video depicts the approach to the lateral sphenoid recess in a patient with CSF rhinorrhea secondary to a lateral sphenoid encephalocele. The transorbital approach represents a unique advantage to repairing the lateral sphenoid sinus in comparison to the endoscopic endonasal approach, which requires traversing, and often transecting, the vidian nerve. Following identification of the skull base defect, repair is performed as an underlay with an abdominal fat graft. Following surgery, the patient had no recurrent rhinorrhea.

The video can be found here: https://stream.cadmore.media/r10.3171/2025.1.FOCVID24207

## Transcript

In this presentation we show a case of transorbital endoscopic approach for repair of a lateral sphenoid sinus CSF leak.

### 0:27 Clinical Presentation.

The patient was a middle-aged woman who presented with recurrent CSF leaks. On exam she had rhinorrhea but was otherwise normal. A thin-cut CT demonstrated a defect in the roof of the lateral sphenoid sinus. We prefer a transorbital approach for these lesions as it provides a direct and short approach to the anterolateral triangle, lateral to the foramen rotundum and medial to the foramen ovale.^1^ Other approaches include the middle fossa craniotomy, which requires elevation and retraction of the temporal lobe, or an endoscopic endonasal transpterygoid approach, which puts the vidian nerve at risk.^2^ For these reasons we prefer the transorbital approach.^3^ During the transorbital approach, care must be taken not to excessively retract the orbit as it can put traction on the optic nerve. In addition, care must be taken during the sphenoid bone drilling not to injure V2 or V3.

### 1:20 Imaging Review.

Axial T2 MRI demonstrates an encephalocele in the location of the anterolateral triangle on the right side. Coronal thin-cut CT demonstrates a dehiscence in the lateral sphenoid sinus roof and demonstrates fluid in the sphenoid sinus on the right side.

### 1:37 Description of Setup.

During the procedure the patient is positioned supine with the head neutral and in Mayfield fixation. Necessary equipment includes neuronavigation, a low-profile high-speed drill, and an endoscope. In the video we will review the key surgical steps which include a transpalpebral eyelid incision, subperiosteal dissection along the lateral orbital rim, retraction of the orbital contents medially, drilling of the lateral orbital wall to expose the temporal dura, release of the meningo-orbital band, resection of the sagittal crest of the greater sphenoid wing, identification of V2 and V3, and repair with abdominal fat and fibrin glue in the anterolateral triangle.

### 2:19 Incision and Dissection.

The skin is prepped with Betadine and local anesthetic with lidocaine and epinephrine is infiltrated. During incision, care is taken not to incise through the orbital septum. A Colorado-tip Bovie is used to define the orbital rim and to incise down to the bone. A subperiosteal dissection is then carried through with a Penfield 1, taking care not to violate the periorbita. The frontozygomatic suture is identified. Retractor is placed medially and the orbital contents are retracted.

### 2:58 Introduction of the Endoscope.

An endoscope is introduced at an approximate 90° angle of entry, and a high-speed, low-profile drill is used to drill the lateral orbital wall, which corresponds to the greater wing of the sphenoid. In this video, the orbit is being retracted medially and is at the top of the screen. The lateral orbital wall is at the bottom of the screen, and to the right of the screen is the feet, and to the left is cranial.

### 3:24 Identification of Sagittal Crest.

As the temporal lobe dura becomes apparent, an island of bone between the dura and periorbita is seen and this corresponds to the sagittal crest of the sphenoid wing. The sagittal crest is a surgically created anatomical landmark. The orbital roof is drilled at its depth and the frontal lobe dura becomes apparent. The remaining lesser sphenoid wing between the temporal lobe and the frontal lobe is then removed and the meningo-orbital band is cauterized and cut. The sagittal crest is being removed here with a Kerrison rongeur.

### 4:12 Cutting the Meningo-Orbital Band.

The face of the greater wing of the sphenoid is drilled down to the floor of the middle fossa. The removal of the sagittal crest allows for retraction of the orbital apex and the cavernous sinus.

### 4:39 Separation of Temporal and Cavernous Sinus Dura.

Using a Freer, the temporal lobe dura is dissected from the lateral dural wall of the cavernous sinus. The camera has been rotated here and the temporal lobe dura is to the left of the screen and the orbit is now to the right. The temporal lobe is elevated from the floor of the middle fossa. The dura was noted to be thin and attenuated here but without any frank defect.

### 5:20 Placement of Abdominal Fat.

Abdominal fat is harvested and is placed overlying the anterolateral triangle.^4,5^ Subsequently, the fibrin glue is used to reinforce the closure.

### 5:31 Closure.

The pericrania is closed along the lateral orbital rim using absorbable sutures in an interrupted fashion. The skin is closed with interrupted 5-0 Prolene sutures.

### 6:00 Disease Background and Postoperative Imaging and Course.

Postoperative MRI demonstrates the placement of the abdominal fat graft. The patient recovered well from surgery and was discharged home on the 1st postoperative day. Sutures were removed on the 5th day after surgery.

## Disclosures

The authors report no conflict of interest concerning the materials or methods used in this study or the findings specified in this publication.

## Author Contributions

Primary surgeon: Schwartz, Godfrey. Assistant surgeon: Delavari. Editing and drafting the video and abstract: Schwartz, Delavari, Lawrence, Testa, Godfrey. Critically revising the work: Schwartz, Delavari, Lawrence, Tangsrivimol, Godfrey. Reviewed submitted version of the work: Schwartz, Delavari, Lawrence, Godfrey. Approved the final version of the work on behalf of all authors: Schwartz. Supervision: Schwartz, Godfrey.

## Supplemental Information

### Patient Informed Consent

The necessary patient informed consent was obtained in this study.
